# Genetic Variation in TLR Genes in Ugandan and South African Populations and Comparison with HapMap Data

**DOI:** 10.1371/journal.pone.0047597

**Published:** 2012-10-24

**Authors:** Allison R. Baker, Feiyou Qiu, April Kaur Randhawa, David J. Horne, Mark D. Adams, Muki Shey, Jill Barnholtz-Sloan, Harriet Mayanja-Kizza, Gilla Kaplan, Willem A. Hanekom, W. Henry Boom, Thomas R. Hawn, Catherine M. Stein, Tuberculosis Research Unit (TBRU) and South African Tuberculosis Vaccine Initiative Team (SATVI)

**Affiliations:** 1 Department of Epidemiology and Biostatistics, Case Western Reserve University, Cleveland, Ohio, United States of America; 2 Department of Genetics and Center for Proteomics and Bioinformatics, Case Western Reserve University, Cleveland, Ohio, United States of America; 3 Case Comprehensive Cancer Center, Case Western Reserve University, Cleveland, Ohio, United States of America; 4 Department of Medicine, Case Western Reserve University, Cleveland, Ohio, United States of America; 5 Department of Medicine, University of Washington School of Medicine, Seattle, Washington, United States of America; 6 Uganda – Case Western Reserve University Research Collaboration, Cleveland, Ohio, United States of America, and Kampala, Uganda; 7 Makerere University School of Medicine and Mulago Hospital, Kampala, Uganda; 8 South African Tuberculosis Vaccine Initiative, Institute of Infectious Diseases and Molecular Medicine and School of Child and Adolescent Health, University of Cape Town, South Africa; 9 Public Health Research Institute, University of Medicine and Dentistry of New Jersey, Newark, New Jersey, United States of America; University of Massachusetts Medical School, United States of America

## Abstract

Genetic epidemiological studies of complex diseases often rely on data from the International HapMap Consortium for identification of single nucleotide polymorphisms (SNPs), particularly those that tag haplotypes. However, little is known about the relevance of the African populations used to collect HapMap data for study populations conducted elsewhere in Africa. Toll-like receptor (TLR) genes play a key role in susceptibility to various infectious diseases, including tuberculosis. We conducted full-exon sequencing in samples obtained from Uganda (n = 48) and South Africa (n = 48), in four genes in the TLR pathway: TLR2, TLR4, TLR6, and TIRAP. We identified one novel TIRAP SNP (with minor allele frequency [MAF] 3.2%) and a novel TLR6 SNP (MAF 8%) in the Ugandan population, and a TLR6 SNP that is unique to the South African population (MAF 14%). These SNPs were also not present in the 1000 Genomes data. Genotype and haplotype frequencies and linkage disequilibrium patterns in Uganda and South Africa were similar to African populations in the HapMap datasets. Multidimensional scaling analysis of polymorphisms in all four genes suggested broad overlap of all of the examined African populations. Based on these data, we propose that there is enough similarity among African populations represented in the HapMap database to justify initial SNP selection for genetic epidemiological studies in Uganda and South Africa. We also discovered three novel polymorphisms that appear to be population-specific and would only be detected by sequencing efforts.

## Introduction

Human genetic studies of diseases with complex inheritance involve analysis of single nucleotide polymorphisms (SNPs) which are present at a range of population-specific frequencies. The proper selection of SNPs for genetic studies requires either discovering the SNPs in the population of interest with *de novo* sequencing efforts or relying on information from similar populations in public databases. The International HapMap Consortium [1], [2] has provided a database of common SNPs in a number of diverse global populations including three from Africa: the Yoruba from Nigeria (YRI), and the Luhya (LWK) and Maasai (MKK) from Kenya. The HapMap project has been instrumental for selection of SNPs for study in a variety of complex diseases in diverse populations. However, the applicability of the data from the African populations described in HapMap to studies in other parts of Africa is less obvious because of the immense genetic diversity on the African continent. Haplotype blocks are shorter in Africans [3], and haplotype and linkage disequilibrium (LD) diversity is abundant [3]–[5]. Thus, studies of genetic variation in other African populations are valuable in understanding how to plan genetic epidemiologic studies in these diverse populations.

Toll-like receptors (TLRs) play a key role in the innate immune response to a variety of pathogens. Mutations and polymorphisms in TLR genes have been associated with susceptibility to various infectious diseases, including Mendelian disorders with mutations in IRAK4, MyD88, TLR3, and Unc93b [6]. In studies of diseases with complex inheritance patterns, TLR polymorphisms have been associated with susceptibility to several infections, including tuberculosis (TB) [7] and leprosy [8]. In addition, previous studies have shown that the TLR1/6/10 region is under natural selection [9]–[11] and that TLR1 has a high degree of population differentiation [8]. Though most studies have focused on common variation in TLR genes, a sequencing study conducted in Houston identified a number of rare variants in TLR genes that were associated with TB [12]. Because of their central role in TB immunity and potential importance for vaccine development, it is of interest to study variants in TLR genes and their association with TB and other infectious diseases in Africa, where they are especially prevalent.

In this study, we conducted full exon sequencing of TLR2, TLR4, TLR6, and TIRAP in samples obtained as part of ongoing studies in Kampala, Uganda, and Cape Town, South Africa. Our objective in this study was first to examine whether there were any novel TLR gene polymorphisms in these study populations, and second, to compare the genotype and haplotype frequencies between these populations and the African HapMap populations.

## Results

To understand haplotype structure and population polymorphism diversity, we sequenced the coding region of four candidate genes in two populations and compared it to four populations of African ancestry in the HapMap database. Specifically, we sequenced gene regions from the Kampala Ugandans (UG, n = 48) and Cape Town South Africans (SA, n = 48) and compared findings to four populations composed of unrelated individuals from the HapMap database: the Maasai in Kinyawa, Kenya (MKK, n = 143), the Luhya in Webuye, Kenya (LWK, n = 90), the African Ancestry in Southwest USA (ASW, n = 53), and Yoruba in Ibadan, Nigeria (YRI, n = 114). Four candidate genes were considered in our analysis: TLR2 (chromosome 4q32), TLR4 (9q32– q33), TLR6 (4p14), and TIRAP (11q23– q24). Genotype frequencies for these populations are provided in Table 1. Only those SNPs genotyped across all six populations were considered in the statistical comparisons. There were two novel polymorphisms discovered in the Ugandan population: one TIRAP SNP (G222A (A74A)) and one TLR6 SNP (A1696G (P564P)). In the South Africans, one novel polymorphism was found in TLR6 (T34A (F12I)), which we previously reported [13]. None of these SNPs were present in the 1000 Genomes database [14]. Of note, TLR2 rs5743709, present in the Ugandan population (MAF 5%) but not in the South African or African HapMap populations, was observed in the 1000 Genomes database in the Asian populations (MAF = 8.8%) and in the Hispanic populations from Puerto Rico and South America (minor allele frequency = 0.2%). We observed departures from Hardy-Weinberg proportions in the Ugandan population in one TLR6 SNP (rs3775073; p = 0.001).

**Table 1:** Genotype frequencies for polymorphisms in TIRAP, TLR2, TLR4, and TLR6 for HapMap populations and South African (SA) and Ugandan (UG) populations, and tests of significance comparing populations.

			HapMap population frequencies				Study pop frequency		p-value comparing all populations	UG vs HapMap p<0.05	SA vs HapMap p<0.05
Gene	rs# bp (aa)	genotype	YRI N = 114	ASW N = 53	LWK N = 90	MKK N = 143	S. AfricaN = 48	UgandaN = 48			
**TIRAP**	rs8177369	CC	–	–	–	–	0.974	0.933	ND	–	–
	C25G	CG	–	–	–	–	0.026	0.067			
	(A9P)	GG	–	–	–	–	0.000	0.000			
	rs8177399	CC	1.000	0.957	–	–	0.947	1.000	ND	–	–
	C37T	CT	0.000	0.043	–	–	0.054	0.000			
	(R13W)	TT	0.000	0.000	–	–	0.000	0.000			
	rs3802813	GG	0.921	0.943	0.811	0.930	0.923	0.851	0.031	None	none
	G164A	AG	0.079	0.057	0.167	0.070	0.077	0.149			
	(S55N)	AA	0.000	0.000	0.022	0.000	0.000	0.000			
	**New**	GG	–	–	–	–	1.000	0.938	ND	–	–
	G222A	AG	–	–	–	–	0.000	0.063			
	(A74A)	AA	–	–	–	–	0.000	0.000			
	rs8177400	GG	–	–	–	–	0.974	0.957	ND	–	–
	G286A	AG	–	–	–	–	0.026	0.043			
	(D96N)	AA	–	–	–	–	0.000	0.000			
	rs3802814	GG	1.000	0.872	–	–	0.897	0.978	ND		
	G303A	AG	0.000	0.128	–	–	0.103	0.022			
	(Q101Q)	AA	0.000	0.000	–	–	0.000	0.000			
	rs74937157	TT	0.988	0.979	0.989	–	1.000	0.958	ND	–	–
	T400C	CT	0.012	0.021	0.011	–	0.000	0.042			
	(C134A)	CC	0.000	0.000	0.000	–	0.000	0.000			
	rs8177374	CC	1.000	0.906	0.989	0.977	0.897	0.978	<0.0001	YRI, LWK, MKK	none
	C539T	CT	0.000	0.094	0.011	0.022	0.103	0.022			
	(S180L)	TT	0.000	0.000	0.000	0.000	0.000	0.000			
	rs7932766	CC	0.746	0.585	0.764	0.594	0.872	0.911	<0.0001	YRI,MKK,ASW	YRI,ASW,MKK
	C558T	CT	0.254	0.396	0.236	0.343	0.103	0.089			
	(A186A)	CC	0.000	0.019	0.000	0.063	0.026	0.000			
	rs7932976	GG	0.920	0.962	0.933	0.979	0.974	0.978	0.12	–	–
	G589A	AG	0.080	0.038	0.067	0.021	0.026	0.022			
	(V197I)	AA	0.000	0.000	0.000	0.000	0.000	0.000			
**TLR2**	rs5743697	CC	1.000	–	–	–	1.000	1.000	ND		
	C114A	AC	0.000	–	–	–	0.000	0.000			
	(G38G)	AA	0.000	–	–	–	0.000	0.000			
	rs3804099	CC	0.412	0.385	0.511	0.385	0.521	0.583	0.03	YRI,ASW, MKK	YRI, ASW,
	C597T	CT	0.430	0.462	0.378	0.503	0.479	0.396			LWK, MKK
	(N199N)	TT	0.158	0.154	0.111	0.112	0.000	0.021			
	rs5743698	GG	1.000	–	–	–	1.000	1.000	ND	–	–
	G639C	CG	0.000	–	–	–	0.000	0.000			
	(L213L)	CC	0.000	–	–	–	0.000	0.000			
	rs5743699	CC	1.000	–	–	–	1.000	1.000	ND	–	–
	C1232T	CT	0.000	–	–	–	0.000	0.000			
	(T411I)	TT	0.000	–	–	–	0.000	0.000			
	rs3804100	TT	0.877	0.887	0.876	0.951	0.938	0.917	0.07	–	–
	T1350C	CT	0.123	0.113	0.124	0.049	0.042	0.062			
	(S450S)	CC	0.000	0.000	0.00	0.000	0.020	0.021			
	rs5743701	CC	–	–	–	–	1.000	0.917	ND	–	–
	C1626G	CG	–	–	–	–	0.000	0.083			
	(L542L)	GG	–	–	–	–	0.000	0.000			
	rs5743703	GG	0.991	–	–	–	0.958	0.917	ND	–	–
	G1736A	AG	0.009	–	–	–	0.042	0.083			
	(R579H)	AA	0.000	–	–	–	0.000	0.000			
	rs5743704	CC	1.000	–	–	–	1.000	1.000	ND	–	–
	C1892A	AC	0.000	–	–	–	0.000	0.000			
	(P631H)	AA	0.000	–	–	–	0.000	0.000			
	rs5743708	GG	1.000	–	–	–	1.000	1.000	ND	–	–
	G2259A	AG	0.000	–	–	–	0.000	0.000			
	(R753Q)	AA	0.000	–	–	–	0.000	0.000			
	rs5743709	GG	1.000	1.000	1.000	–	1.000	0.895	ND	–	–
	G2343A	AG	0.000	0.000	0.000	–	0.000	0.105			
	(A781A)	AA	0.000	0.000	0.000	–	0.000	0.000			
**TLR4**	rs2770150	AA	0.789	0.792	0.756	0.720	0.737	0.711	0.46	–	–
		AG	0.211	0.208	0.222	0.231	0.263	0.263			
		GG	0.000	0.000	0.022	0.049	0.000	0.026			
	rs10759931	GG	–	–	–	–	0.921	0.816	ND	–	–
		AG	–	–	–	–	0.079	0.184			
		AA	–	–	–	–	0.000	0.000			
	rs4986790	AA	0.920	0.906	0.807	0.838	1.000	0.921	0.009	None	LWK, MKK
	A896G	AG	0.080	0.075	0.193	0.155	0.000	0.079			
	(D299G)	GG	0.000	0.019	0.000	0.007	0.000	0.000			
	rs4986791	CC	1.000	–	–	–	1.000	1.000	ND	–	–
	C1196T	CT	0.000	–	–	–	0.000	0.000			
	(T399I)	TT	0.000	–	–	–	0.000	0.000			
	rs5030719	GG	0.938	0.943	0.833	0.923	1.000	1.000	0.021	LWK	LWK
	G1530T	GT	0.062	0.057	0.156	0.070	0.000	0.000			
	(Q510H)	TT	0.000	0.000	0.011	0.007	0.000	0.000			
	rs11536889	GG	1.000	–	–	–	1.000	1.000	ND	–	–
		GC	0.000	–	–	–	0.000	0.000			
		CC	0.000	–	–	–	0.000	0.000			
	rs7873784	GG	0.527	–	–	–	0.737	0.711	ND	–	–
		GC	0.382	–	–	–	0.263	0.237			
		CC	0.091	–	–	–	0.000	0.052			
**TLR6**	**new**	TT					0.590	1.000	ND	–	–
	T34A	AT					0.360	0.000			
	(F12I)	AA					0.050	0.000			
	rs5743808	AA	0.851	0.660	0.656	0.888	0.854	0.958	<0.001	ASW, LWK,	ASW, LWK
	A359G	AG	0.131	0.321	0.311	0.112	0.146	0.021		MKK	
	(I120T)	GG	0.018	0.019	0.033	0.000	0.000	0.021			
	rs5743809	TT	0.927	–	–	–	0.920	0.980	ND	–	–
	T581C	CT	0.073	–	–	–	0.080	0.020			
	(L194P)	CC	0.000	–	–	–	0.000	0.000			
	rs35220466	GG	0.950	–	–	–	0.940	0.770	ND	–	–
	G740A	AG	0.050	–	–	–	0.060	0.030			
	(R247K)	AA	0.000	–	–	–	0.000	0.200			
	rs5743810	TT	1.000	–	–	–	0.766	0.875	ND	–	–
	T745C	CT	0.000	–	–	–	0.020	0.125			
	(S249P)	CC	0.000	–	–	–	0.030	0.000			
	rs3796508	CC	0.965	0.830	0.944	0.951	0.958	1.000	.012	ASW	
	C979T	CT	0.035	0.170	0.056	0.049	0.042	0.000			
	(V327M)	TT	0.000	0.000	0.000	0.000	0.000	0.000			
	rs3821985	CC	0.463	–	–	–	0.210	0.960	ND	–	–
	C1083G	CG	0.444	–	–	–	0.340	0.040			
	(T361T)	GG	0.093	–	–	–	0.450	0.000			
	rs3775073	CC	0.579	0.434	0.708	0.448	0.479	0.730	<0.001	YRI, MKK,	LWK
	C1263TT	CT	0.351	0.491	0.270	0.455	0.333	0.104		LWK, ASW	
	(K421K)	TT	0.070	0.075	0.022	0.098	0.188	0.167			
	rs5743815	TT	1.000	–	–	–	0.990	1.000	ND	–	–
	T1280C	CT	0.000	–	–	–	0.010	0.000			
	(V427A)	CC	0.000	–	–	–	0.000	0.000			
	rs5743816	GG	0.982	–	–	–	–	0.900	ND	–	–
	G1393A	AG	0.018	–	–	–	–	0.100			
	(V465I)	AA	0.000	–	–	–	–	0.000			
	**new**	AA	–	–	–	–	–	0.920	ND	–	–
	A1696G	AG	–	–	–	–	–	0.080			
	(P564P)	GG	–	–	–	–	–	0.000			
	rs5743818	TT	–	–	–	–	–	0.980	ND	–	–
	T1932G	GT	–	–	–	–	–	0.020			
	(A644A)	GG	–	–	–	–	–	0.000			

ASW = African ancestry in Southwest USA; YRI = Yoruba in Ibadan, Nigeria; LWK = Luhya in Webuye, Kenya; MKK = Maasai in Kinyawa, Kenya; UG = Ugandan from Kampala; SA = South Africans from Cape Town.

SNPs not included in the HapMap are denoted by “-“ and were not included in statistical comparisons, as denoted by “ND” in the table.

Novel polymorphisms denoted by “new”. Beneath rs number is the base pair numbering for coding region polymorphisms based on a system when the start codon ATG = 1. Amino acid numbering is listed below the base pair in parentheses.

SNPs where common homozygote was at 100% frequency in all populations have been excluded from this table.

Comparison of genotype frequencies across these six populations suggested that there were a range of frequencies that were often different among the groups (p<0.05 by chi-square test) (Table 1). When comparing our sequencing results to the HapMap populations, the most significant differences were seen when comparing UG to the HapMap population, especially within the TLR6 gene. SA, as a whole, showed fewer significant differences from the HapMap populations. P-values for all pair-wise comparisons are given in Table 1. Heterozygosity values for each SNP in each population are provided in Table S1. The ratio of observed to expected heterozygosity was generally similar across populations. The notable exception to this was TLR6 in the Ugandan population, where the ratio of observed to expected heterozygosity was very low (0.216), which reflects decreased genetic diversity. Also, TLR4 shows reduced genetic diversity in both SA and UG, reflecting the presence of monomorphic SNPs in these populations.

**Table 2:** TLR2 Haplotype Comparison Among Populations.

		HAPLOTYPE			
		C-C	C-T	T-T	T-C
**Population**	**LWK**	0.06110	0.6389	0.3000	–
	**MKK**	0.02045	0.6170	0.3626	–
	**YRI**	0.06023	0.5663	0.3735	–
	**UG**	0.2857	0.5000	–	0.2143
	**SA Mixed**	0.5039	0.2688	0.2273	–

SNPs in haplotype: rs3804099–rs3804100.

We next examined LD patterns and haplotype structure between the different groups (Table S2). In these and subsequent analyses, the SA population was stratified into its component ethnic groups: Black, Caucasian, and South African Mixed Ethnicity. Both the UG and SA Mixed Ethnicity population, as well as the HapMap populations, showed low amounts of LD (absolute value of r^2^<0.2 for 76.9% of comparisons). Haplotypes were constructed for the SNPs that were common between our sequencing study and HapMap, and haplotype frequencies were compared between the populations (Tables 2, 3, 4, 5). For this analysis, we considered only the African populations, UG, SA Mixed, MKK, LWK, and YRI, choosing not to include the American individuals with African ancestry (ASW) as our goal was to identify which African populations are “similar” to the UG and SA Mixed individuals. Also excluded from the haplotype analyses were the Black and Caucasian South Africans due to limited sample size. When comparing across all five populations, there were significant differences in haplotype frequencies for all genes (p<0.0001). The most unique result from these analyses was that haplotypes in TLR2 and TLR6 in the UG individuals were absent from the other five populations. Also, some rarer haplotypes (less than 10% frequency) were unique to some HapMap populations. Analyses of TLR6 haplotype frequencies were not feasible as the UG haplotypes did not exist in the other populations (Tables 2, 3, 4, 5).

**Table 3:** TLR4 Haplotype Comparison Among Populations.

		HAPLOTYPE						
		A-A-G	A-A-T	A-G-G	A-G-T	G-A-G	G-G-G	G-A-T
**Population**	**LWK**	0.7555	0.01725	0.02782	0.06609	0.1266	0.001183	0.005556
	**MKK**	0.7714	–	0.03292	0.03193	0.1464	0.01422	0.003045
	**YRI**	0.8510	0.01058	0.03032	0.02071	0.08205	–	0.005004
	**UG**	0.8026	–	0.03947	–	0.1579	–	–
	**SA Mixed**	0.8462	–	–	–	0.1538	–	–

SNPs in haplotype: rs2770150–rs4986790–rs5030719.

**Table 4:** TLR6 Haplotype Comparison Among Populations.

		HAPLOTYPE								
		C-C-A	C-C-G	T-C-A	C-T-G	T-C-G	T-G-A	C-G-A	T-G-G	C-G-G
**Population**	**LWK**	0.6545	0.1611	0.1566	0.0278	–	–	–	–	–
	**MKK**	0.6184	0.0249	0.3290	0.0234	0.0043	–	–	–	–
	**YRI**	0.6898	0.0572	0.2379	0.0151	–	–	–	–	–
	**UG**	–	–	–	–	–	0.6771	0.2917	0.0208	0.0104
	**SA Mixed**	0.5978	0.0581	0.3113	0.0326	–	–	–	–	–

SNPs in haplotype: rs3775073–rs3796508–rs574–3808.

**Figure 1:**
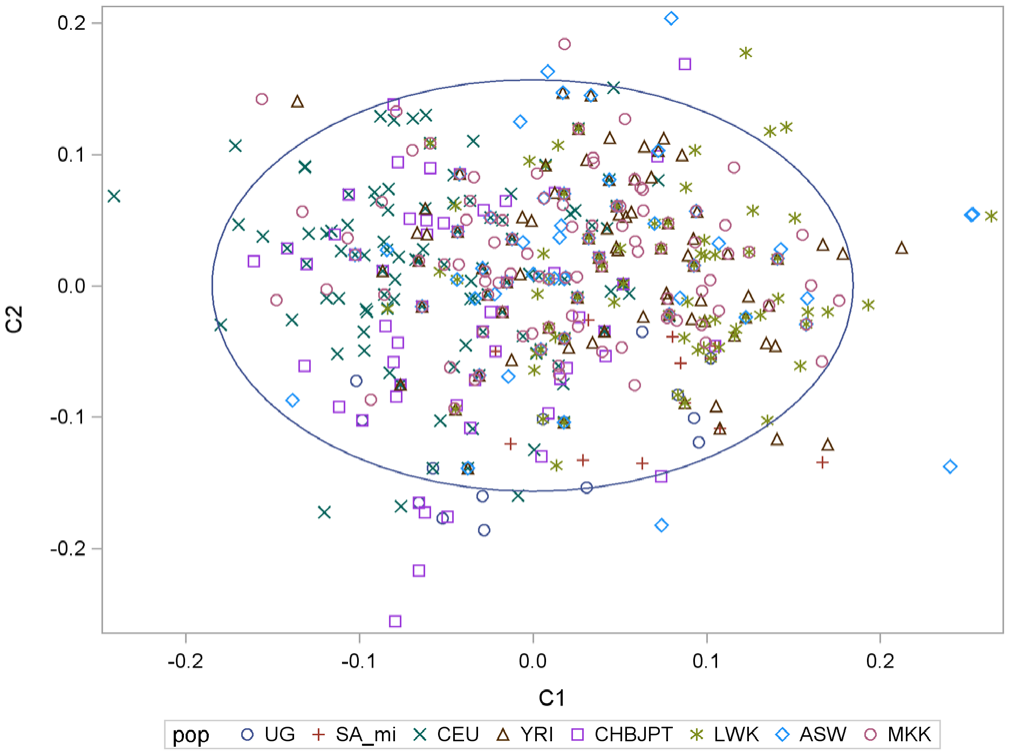
Plot of first two dimensions from MDS analysis.

Multidimensional scaling (MDS) analysis, which combined the TLR2, TLR4, TLR6, and TIRAP data, was used to illustrate how UG and SA Mixed Ethnicity populations clustered with the HapMap populations. We plotted the first two dimensions from MDS (Figure 1). Visual examination of this plot shows a great deal of overlap among these African populations as well as with the ASW, with a few outlying points. The SA Mixed Ethnicity population tended to cluster more with the ASW population, as well as with the MKK, LWK, and YRI. The UG population primarily clustered with the MKK, LWK, and YRI. Analysis of Euclidean distances between individuals within different population clusters showed that distances between individuals were not significantly different (all pairwise p-values >0.27); this suggests that there is overlap among all the population clusters.

**Table 5:** TIRAP Haplotype Comparison Among Populations.

		HAPLOTYPE							
		A-C-G-C	G-C-A-C	G-C-G-C	G-T-G-C	G-C-G-T	G-T-A-C	A-C-A-C	A-T-G-C
**Population**	**LWK**	0.1042	0.0299	0.7411	0.1157	0.0057	0.0021	0.0014	–
	**MKK**	0.0347	0.0087	0.6810	0.2216	0.0505	–	–	0.0034
	**YRI**	0.0362	0.0361	0.7908	0.1334	–	–	–	0.0029
	**UG**	0.0778	0.0111	0.8556	0.0444	0.0111	–	–	–
	**SA Mixed**	0.0313	0.0156	0.8281	0.0625	0.0625	–	–	–

SNPs in haplotype: rs3802813–rs7932766–rs7932976–rs8177374.

## Discussion

The primary finding of our study was that the genotype frequency and haplotype structure of Ugandans and South Africans of Mixed ethnicity are similar to those in the HapMap database among the Kenyan and Yoruba Nigerian reference populations. A practical issue for genetic association studies is to determine whether tag SNPs identified using HapMap data adequately capture patterns of variation in other populations [15]. Thus, it is of interest to examine both genotype frequencies and haplotype frequencies between HapMap populations and other global populations. Genetic diversity, measured by the ratio of observed to expected heterozygosity, was also generally similar across these populations. Examination of haplotype frequencies and LD patterns is also informative for identification of the appropriate population(s) for tag SNP selection [16]. Generally, our data showed the most common haplotype in each of the four genes was the same across all the populations, though the less common haplotypes differed and there were some unique haplotypes in the Ugandan population. We observed notable haplotype frequency differences between the Ugandan population and the HapMap populations in TLR6 (discussed in depth below) and TLR2, showing that differences between African populations do exist and tag SNPs should be selected judiciously. Though there were differences in haplotype frequencies across populations, we also observed overlap among populations in our cluster analysis. This latter analysis is only exploratory, since it is based on polymorphisms common to exons and HapMap in four genes. When the African data are examined as a whole, there is notable similarity, though there are slight differences between pairs of populations.

Many studies [9], [15] have suggested that genetic similarity between populations is generally predictable based on geographic location. Conrad et al. [15] concluded that HapMap is indeed a valuable resource, and geography could be used to identify the most appropriate HapMap population because haplotype similarity is greatest in nearby populations. However, that study was conducted prior to the release of HapMap Release 3 data, which included many new reference populations, including the Kenyan groups (MKK and LWK). If we examine our haplotype data, geographic and genetic similarity were less evident when comparing the Ugandan population with the three African populations in the HapMap database. For example, for TLR2, some of the Ugandan haplotype frequencies were intermediate between the Kenyan LWK/MKK and the Nigerian YRI, while other haplotype frequencies were similar with LWK/MKK or YRI. TIRAP is interesting in that MKK and YRI were more similar to each other than MKK is to LWK. Since MKK and LWK are geographically near each other, this may seem surprising, but the Luhya and Maasai tribal histories are quite different. The Ugandan haplotypes showed similarity to all three populations. Together our data suggests that geographic proximity is only a partial predictor of the haplotype structure of a gene in different populations.

Another noteworthy finding of our study was that a small number of novel polymorphisms were detected in TLR6 and TIRAP. The relevance of rare variants in complex trait susceptibility is gaining attention [17]. Ma et al. [12] also conducted sequencing of TLR genes and observed there were more rare non-synonymous polymorphisms in African-American and Caucasian TB cases than in controls. In addition, they found that rare variants were overrepresented in the TLR1/6/10 region. Our findings support a conclusion of Ma et al. that resequencing strategies are valuable in the search for rare and population-specific variants that may be associated with disease, particularly in populations of African descent.

The occurrence of novel polymorphisms, such as in the Ugandan population on TLR6, results in unique haplotypes not seen in other populations, which is consistent with a potential effect of selection [18]. There is additional evidence of positive selective pressure on TLR6 in the Ugandan population. One TLR6 SNP in the Ugandan population is in significant deviation from Hardy-Weinberg proportions. The existence of a unique, common polymorphism (A1696G) and significant shift in genotype frequencies (rs3775073 and rs3821985) are additional indicators [5]. There is also significantly reduced heterozygosity in TLR6 in the Ugandan population, further reflecting selective pressure [19]. The novel TLR6 SNP in the South African population (T34A) is also quite common. Previous studies have shown that the TLR1/6/10 region is under natural selection [9]–[11]. There is also a unique, common TLR2 haplotype in the Ugandan population, suggesting selective effects on TLR2. As suggested by Barreiro and Quintana-Murci [5], complex traits like TB are likely polygenic, so the effects of selection on individual loci are likely weaker. Actual population genetic tests examining effects of selection require full sequence data, so are beyond the scope of this paper.

There are a few limitations with this analysis. We restricted our analysis to TLR pathway genes, because of their key role in the innate immune response. Generalizations to the rest of the genome cannot be made based on only four genes, and selective pressure on immunity genes may result in different population genetic parameters than the rest of the genome. Second, our haplotype and LD analyses were restricted to SNPs that were common to both our exomic sequencing efforts and the HapMap. Furthermore, some SNPs were represented in Phase I and II of the HapMap, but not Phase III, and vice versa. Because of the differences seen in haplotype frequencies and LD, some information may have been lost by virtue of this aspect of study design. Finally, our sample size was underpowered to detect small differences between populations. We had 70–80% power to detect a difference of 0.2 of allele frequencies, but only had 20–30% power to detect differences of 0.1.

In conclusion, we found that there is more similarity across African populations than there is dissimilarity, though patterns of similarity do not necessarily reflect geographic proximity. Thus, HapMap provides a good starting point for genetic association studies. However, one must be mindful of possible LD differences between specific populations and those represented on the HapMap. Selective pressure by TB and other infectious diseases may have influenced differential LD structure across Africa. For this reason, we suggest using all three African HapMap populations as the reference for tag SNP selection, as has been advocated by other studies [20]. Since it is well-known that African populations show such high genetic diversity, unique polymorphisms may exist in those populations that may not be represented in the HapMap panels. Thus, follow-up sequencing of certain genes may be warranted in specific populations. Our findings also have utility for admixture mapping studies, which require data on ancestral populations [21].

## Materials and Methods

### Study population

Samples were obtained as part of two ongoing studies in Uganda and South Africa. Ugandan samples were initially collected as part of the Household Contact Study [22] and Kawempe Community Health Study [23], both of which enrolled individuals from urban Kampala, Uganda. For this sequencing study, we selected 48 unrelated healthy individuals who were part of a whole genome scan study [24]. Most of these individuals (87.5%) self-identified their tribe as Baganda; the remaining identified themselves as Rwandese (2 individuals), Zairean, Nubian, Langi, and Acholi. An analysis of substructure using STRUCTURE in our genome scan data showed that there was no substructure within the larger dataset [24], so we analyzed all of the Ugandan individuals together.

The South African samples were collected from healthy adults enrolled at the South African Tuberculosis Vaccine Initiative clinical site near Cape Town in South Africa [13]. Exclusion criteria included HIV or other chronic infections, pregnancy or active tuberculosis. The study population included individuals from different backgrounds, including Black African (n = 8), Caucasian (n = 7) and South African Mixed Ethnicity (n = 33). The latter is a distinct group that emerged more than 300 years ago and received genetic influences from Malaysia, Indonesia, European Caucasoid and Black Africans [21], [25].

### Ethics statement

The institutional review boards at University Hospitals of Cleveland and the Uganda Council for Science and Technology approved the Ugandan study. All individuals in the Ugandan study provided written informed consent. For the South African study, all protocols for this study were approved by the Research Ethics Committee of the University of Cape Town and the Institutional Review Boards at the University of Washington and University of Medicine and Dentistry of New Jersey. Ethical guidelines of the US Department of Health and Human Services and the South African Medical Research Council were adhered to, including written informed consent from parents of study participants.

### Genomic methods

Genomic DNA was purified from peripheral blood by QIAamp DNA blood kit (Qiagen). We sequenced the coding region to look for polymorphisms and obtained high quality sequence of the entire coding region in 96 subjects for TLR2 (HapMap Genome Browser Release #28, genomic region coordinates Chromosome 4q32∶154,824,891 to 154,846,690), 76 for TLR4 (Chromosome 9q32–q33∶119,506,431 to 119,519,585), 96 for TLR6 (Chromosome 4p14∶38,504,803 to 38,507,555), and 87 for TIRAP (Chromosome 11q23–q24∶125,658,192 to 125,668,281). The coding region was amplified by PCR, sequenced with Big Dye Terminator v3.0, and analyzed on an ABI PRISM 3730 capillary sequencer (Applied Biosystems). Sequence was aligned and analyzed with the programs PHRED/PHRAP and CONSED [26], [27]. Only individuals with high quality sequence throughout the entire coding region were included in the analysis. An initial PHRED quality value (q) of 30 was used to automatically screen for potential polymorphisms (corresponds to an error rate of 1/1000). All potential polymorphisms were assessed with manual inspection of the chromatogram and then confirmed with sequence obtained in forward and reverse directions. The coding region length, number of coding region exons, and PCR primers for each gene were: TLR2∶2354 base pairs, one exon, 2 amplification products with primers TH20/24 and TH28/31; TLR4∶2520 base pairs, three exons, 3 amplification products T4-11/T4-41 (Exon I), T4-2/T4-12 (Exon III), T4-3/T4-15 (Exon IV); TLR6∶2390 base pairs, one exon, 2 amplification products with primers T6-2/T6-18 and T6-3/T6-16; TIRAP 707 base pairs, 2 exons, 2 amplification products with primers TIRAP-4/21 (Exon I) and TIRAP-3/23 (Exon II). The PCR primer sequences were: TH20 5′TCCATTTTTCAGAACTATCCACTGG3’; TH24 5′TCCTCAAATGACGGTACATCCACG3’; TH28 5′CATAACCTGAAACAAACTTTCATCGG3’, TH31 5′GGTCCCAAAGCATGCTACTCCTGG3’, T4-2 5′GGAAGGATGGACAGATGGATGAAAGG3’, T4-3 5′CCCATCACATCTGTATGAAGAGCTGG3’, T4-11 5′CAGGGCACACAGTGAGAAGTTCTGGGC3’, T4-12 5′GGAGAATAGAGGTAGCTTGCTCAAGG3’, T4-15 5′GCAGCCCTGCATATCTAGTGCACCATGG-3′, T4-41 5′CTTTAGCCACTGGTCTGCAGGCG3’, T6-2 5′GTGGAGGTTTGAGAGTAACCATCCG3’,T6-3 5′CACATGCTGTGTCCTCATGCACCAAGC3’, T6-16 5′GGCTAACCTCACCGCCTAGCTCAGTTCCCC3’, T6-18 5′GGCATATCCTTCGTCATGAGACC-3′, TIRAP-3 5′GTGGAGCAACAGGTCTCTGAGAATAAGATG3’, TIRAP-4 5′GAATGAGAGCAGGGTAAGTGCAGCCTTTGTG3’, TIRAP-21 5′GCGTCTCTCTGAGTTTGGACC3’, TIRAP-23 5′CCAAGGCACAGAGCGGGTGAGTAACTTGG3’.

### Data analysis

Hardy-Weinberg proportions were tested within healthy populations for each SNP using HWSIM (http://krunch.med.yale.edu/hwsim/) with 10,000 iterations. Genotype frequencies were compared across groups using a chi-squared test (or Fisher’s exact test when appropriate) in SAS PROC FREQ. LD was assessed using Haploview software, calculating both r^2^ and D’. Haplotypes were estimated using DECIPHER (S.A.G.E. version 6.0), using the most likely phase for each individual. Differences in haplotype frequencies across populations were evaluated using chi-square or Fisher’s exact tests, as well as for the genotype comparison tests.

To examine how our populations clustered with the HapMap populations, we conducted multidimensional scaling (MDS) using PLINK (http://pngu.mgh.harvard.edu/purcell/plink/). MDS is similar to principal components analysis in that it utilizes SNP genotype data to estimate a matrix of allele sharing identical by state (IBS) and constructs a similarity matrix, then represents each subject by a vector of coordinates [28]. We plotted the first two dimensions to assess how the Ugandan (UG) and South African Mixed (SA Mixed) individuals clustered with the other populations, using MDS in the same way that it is used for identifying population stratification. In order to quantitatively assess the overlap between these populations, we estimated the Euclidean distance between each individual. These distances were used to construct a distribution, which was approximately normal. Then, we estimated the average distance between members of two populations (UG, SA Mixed, etc.) and assessed if the difference was statistically significant using the normal distribution.

## Supporting Information

Table S1 **Heterozygosity values for each SNP, gene, and averaged over all SNPs, by population.**
(DOC)

Table S2 **Pairwise linkage disequilibrium (D’ and r^2^) for each gene (TLR2, TLR4, TLR6, and TIRAP) for the Ugandan, South African, and HapMap populations.**
(DOC)
