# Supplementary material for: Genetic Variation in TLR Genes in Ugandan and South African Populations and Comparison with HapMap Data
Source: PLoS One. 2012 Oct 24;7(10):e47597. doi: 10.1371/journal.pone.0047597 (PMC3480404; doi:10.1371/journal.pone.0047597)
Supplement: Table S1 — Heterozygosity values for each SNP, gene, and averaged over all SNPs, by population. (DOC) [file pone.0047597.s001.doc]

**Supplemental Table 2.** Heterozygosity values for each SNP, gene, and averaged over all SNPs, by population

|  |  | **Population** | | | | | |
| --- | --- | --- | --- | --- | --- | --- | --- |
|  |  | **ASW** | **LWK** | **MKK** | **YRI** | **SA** | **UG** |
| **TIRAP** |  |  |  |  |  |  |  |
| rs3802813 | He | 0.0550 | 0.1888 | 0.0675 | 0.0758 | 0.0740 | 0.1378 |
|  | Ho | 0.0566 | 0.1667 | 0.0699 | 0.0789 | 0.0769 | 0.1489 |
|  | ratio | 1.0291 | 0.8826 | 1.0362 | 1.0411 | 1.0400 | 1.0805 |
| rs7932766 | He | 0.3398 | 0.2081 | 0.3588 | 0.2220 | 0.1420 | 0.0849 |
|  | Ho | 0.3962 | 0.2360 | 0.3427 | 0.2544 | 0.1026 | 0.0889 |
|  | ratio | 1.1661 | 1.1338 | 0.9551 | 1.1457 | 0.7222 | 1.0465 |
| rs7932776 | He | 0.0370 | 0.0644 | 0.0209 | 0.0771 | 0.0253 | 0.0220 |
|  | Ho | 0.0377 | 0.0667 | 0.0211 | 0.0804 | 0.0256 | 0.0222 |
|  | ratio | 1.0192 | 1.0345 | 1.0107 | 1.0419 | 1.0130 | 1.0112 |
| rs8177374 | He | 0.0899 | 0.0113 | 0.0226 | 0.0000 | 0.0973 | 0.0220 |
|  | Ho | 0.0943 | 0.0114 | 0.0229 | 0.0000 | 0.1026 | 0.0222 |
|  | ratio | 1.0495 | 1.0057 | 1.0116 | 0.0000 | 1.0541 | 1.0112 |
| **Average ratio** |  | **1.0660** | **1.0141** | **1.0034** | **0.8072** | **0.9573** | **1.0374** |
| **TLR2** |  |  |  |  |  |  |  |
| rs3804099 | He | 0.4734 | 0.4200 | 0.4628 | 0.4676 | 0.3644 | 0.3418 |
|  | Ho | 0.4615 | 0.3778 | 0.5035 | 0.4298 | 0.4792 | 0.3958 |
|  | ratio | 0.9750 | 0.8995 | 1.0879 | 0.9191 | 1.3151 | 1.1581 |
| rs3804101 | He | 0.1068 | 0.1160 | 0.0478 | 0.1153 | 0.0799 | 0.0987 |
|  | Ho | 0.1132 | 0.1236 | 0.0490 | 0.1228 | 0.0417 | 0.0625 |
|  | ratio | 1.0600 | 1.0659 | 1.0251 | 1.0654 | 0.5217 | 0.6330 |
| **Average ratio** |  | **1.0175** | **0.9827** | **1.0565** | **0.9923** | **0.9184** | **0.8955** |
| **TLR4** |  |  |  |  |  |  |  |
| rs2770150 | He | 0.1860 | 0.2311 | 0.2747 | 0.1884 | 0.2285 | 0.2659 |
|  | Ho | 0.2075 | 0.2222 | 0.2308 | 0.2105 | 0.2632 | 0.2632 |
|  | ratio | 1.1158 | 0.9615 | 0.8402 | 1.1176 | 1.1515 | 0.9896 |
| rs4986790 | He | 0.1068 | 0.1745 | 0.1547 | 0.0765 | 0.0000 | 0.0758 |
|  | Ho | 0.0755 | 0.1932 | 0.1549 | 0.0796 | 0.0000 | 0.0789 |
|  | ratio | 0.7067 | 1.1069 | 1.0013 | 1.0415 | 0.0000 | 1.0411 |
| rs5030719 | He | 0.0550 | 0.1620 | 0.0804 | 0.0600 | 0.0000 | 0.0000 |
|  | Ho | 0.0566 | 0.1556 | 0.0699 | 0.0619 | 0.0000 | 0.0000 |
|  | ratio | 1.0291 | 0.9604 | 0.8698 | 1.0320 | 0.0000 | 0.0000 |
| **Average ratio** |  | **0.9505** | **1.0096** | **0.9038** | **1.0637** | **0.3838** | **0.6769** |
| **TLR6** |  |  |  |  |  |  |  |
| rs3775073 | He | 0.4357 | 0.2651 | 0.4389 | 0.3706 | 0.4575 | 0.3418 |
|  | Ho | 0.4906 | 0.2697 | 0.4545 | 0.3509 | 0.3333 | 0.1042 |
|  | ratio | 1.1258 | 1.0171 | 1.0357 | 0.9468 | 0.7287 | 0.3048 |
| rs3796508 | He | 0.1554 | 0.0546 | 0.0478 | 0.0345 | 0.0408 | 0.0000 |
|  | Ho | 0.1698 | 0.0562 | 0.0490 | 0.0351 | 0.0417 | 0.0000 |
|  | ratio | 1.0928 | 1.0289 | 1.0251 | 1.0179 | 1.0213 | 0.0000 |
| rs5743808 | He | 0.2942 | 0.3064 | 0.1056 | 0.1528 | 0.1352 | 0.0605 |
|  | Ho | 0.3208 | 0.3111 | 0.1119 | 0.1316 | 0.1458 | 0.0208 |
|  | ratio | 1.0901 | 1.0153 | 1.0593 | 0.8612 | 1.0787 | 0.3441 |
| **Average ratio** |  | **1.1029** | **1.0205** | **1.0400** | **0.9420** | **0.9429** | **0.2163** |
|  |  |  |  |  |  |  |  |
| **Overall** | **He** | **0.1946** | **0.1835** | **0.1735** | **0.1534** | **0.1371** | **0.1209** |
|  | **Ho** | **0.2067** | **0.1825** | **0.1733** | **0.1530** | **0.1344** | **0.1006** |
|  | **ratio** | **1.0622** | **0.9943** | **0.9989** | **0.9975** | **0.9804** | **0.8321** |

He = observed heterozygosity (proportion of heterozygous genotypes), He = expected heterozygosity (1-p^2^-q^2^), ratio = Ho/He
