# Supplementary material for: Genetic Variation in TLR Genes in Ugandan and South African Populations and Comparison with HapMap Data
Source: PLoS One. 2012 Oct 24;7(10):e47597. doi: 10.1371/journal.pone.0047597 (PMC3480404; doi:10.1371/journal.pone.0047597)
Supplement: Table S2 — Pairwise linkage disequilibrium (D’ and r2) for each gene (TLR2, TLR4, TLR6, and TIRAP) for the Ugandan, South African, and HapMap populations. (DOC) [file pone.0047597.s002.doc]

**Supplemental material. Linkage disequilibrium (LD), in terms of D’ and r^2^, by gene and population**

| **TIRAP** | **UG** |  |  |  |  | **TIRAP** | **SA** |  |  |  |
| --- | --- | --- | --- | --- | --- | --- | --- | --- | --- | --- |
| D' |  |  |  |  |  | D' |  |  |  |  |
|  | rs3802813 | rs8177374 | rs7932766 | rs7932976 |  |  | rs3802813 | rs8177374 | rs7932766 | rs7932976 |
| rs3802813 | - | 1.000 | 1.000 | 1.000 |  | rs3802813 | - | 1.000 | 1.000 | 1.000 |
| rs8177374 | | - | 1.000 | 1.000 |  | rs8177374 | | - | 1.000 | 1.000 |
| rs7932766 | |  | - | 1.000 |  | rs7932766 | |  | - | 1.000 |
| rs7932976 | |  |  | - |  | rs7932976 | |  |  | - |
|  |  |  |  |  |  |  |  |  |  |  |
| R^2^ |  |  |  |  |  | R^2^ |  |  |  |  |
|  | rs3802813 | rs8177374 | rs7932766 | rs7932976 |  |  | rs3802813 | rs8177374 | rs7932766 | rs7932976 |
| rs3802813 | - | 0.001 | 0.004 | 0.001 |  | rs3802813 | - | 0.002 | 0.002 | 0.001 |
| rs8177374 | | - | 0.001 | 0.000 |  | rs8177374 | | - | 0.004 | 0.001 |
| rs7932766 | |  | - | 0.001 |  | rs7932766 | |  | - | 0.001 |
| rs7932976 | |  |  | - |  | rs7932976 | |  |  | - |
|  |  |  |  |  |  |  |  |  |  |  |
| **TIRAP** | **MKK** |  |  |  |  | **TIRAP** | **LWK** |  |  |  |
| D' |  |  |  |  |  | D' |  |  |  |  |
|  | rs3802813 | rs8177374 | rs7932766 | rs7932976 |  |  | rs3802813 | rs8177374 | rs7932766 | rs7932976 |
| rs3802813 | - | 1.000 | 0.454 | 1.000 |  | rs3802813 | - | 1.000 | 1.000 | 0.499 |
| rs8177374 | | - | 0.999 | 1.000 |  | rs8177374 | | - | 1.000 | 1.000 |
| rs7932766 | |  | - | 0.833 |  | rs7932766 | |  | - | 0.366 |
| rs7932976 | |  |  | - |  | rs7932976 | |  |  | - |
|  |  |  |  |  |  |  |  |  |  |  |
| R^2^ |  |  |  |  |  | R^2^ |  |  |  |  |
|  | rs3802813 | rs8177374 | rs7932766 | rs7932976 |  |  | rs3802813 | rs8177374 | rs7932766 | rs7932976 |
| rs3802813 | - | 0.002 | 0.002 | 0.000 |  | rs3802813 | - | 0.001 | 0.016 | 0.001 |
| rs8177374 | | - | 0.015 | 0.001 |  | rs8177374 | | - | 0.001 | 0.000 |
| rs7932766 | |  | - | 0.002 |  | rs7932766 | |  | - | 0.001 |
| rs7932976 | |  |  | - |  | rs7932976 | |  |  | - |
| **TIRAP** | **ASW** |  |  |  |  | **TIRAP** | **YRI** |  |  |  |
| D' |  |  |  |  |  | D' |  |  |  |  |
|  | rs3802813 | rs8177374 | rs7932766 | rs7932976 |  |  | rs3802813 | rs8177374 | rs7932766 | rs7932976 |
| rs3802813 | - | 0.264 | 1.000 | 1.000 |  | rs3802813 | - | 1.000 | 0.109 | 1.000 |
| rs8177374 | | - | 0.203 | 1.000 |  | rs8177374 | | - | 1.000 | 1.000 |
| rs7932766 | |  | - | 0.129 |  | rs7932766 | |  | - | 0.373 |
| rs7932976 | |  |  | - |  | rs7932976 | |  |  | - |
|  |  |  |  |  |  |  |  |  |  |  |
| R^2^ |  |  |  |  |  | R^2^ |  |  |  |  |
|  | rs3802813 | rs8177374 | rs7932766 | rs7932976 |  |  | rs3802813 | rs8177374 | rs7932766 | rs7932976 |
| rs3802813 | - | 0.041 | 0.009 | 0.001 |  | rs3802813 | - | -1.000 | 0.003 | 0.002 |
| rs8177374 | | - | 0.001 | 0.001 |  | rs8177374 | | - | -1.000 | -1.000 |
| rs7932766 | |  | - | 0.001 |  | rs7932766 | |  | - | 0.001 |
| rs7932976 | |  |  | - |  | rs7932976 | |  |  | - |
|  |  |  |  |  |  |  |  |  |  |  |
| **TLR6** | **UG** |  |  |  |  | **TLR6** | **SA** |  |  |  |
| D' |  |  |  |  |  | D' |  |  |  |  |
|  | rs5743808 | rs3796508 | rs3775073 |  |  |  | rs5743808 | rs3796508 | rs3775073 |  |
| rs5743808 | - | 1.000 | 0.045 |  |  | rs5743808 | - | 1.000 | 1 |  |
| rs3796508 | | - | 1.000 |  |  | rs3796508 | | - | 0.800 |  |
| rs3775073 | |  | - |  |  | rs3775073 | |  | - |  |
|  |  |  |  |  |  |  |  |  |  |  |
| R^2^ |  |  |  |  |  | R^2^ |  |  |  |  |
|  | rs5743808 | rs3796508 | rs3775073 |  |  |  | rs5743808 | rs3796508 | rs3775073 |  |
| rs5743808 | - | -1.000 | 0 |  |  | rs5743808 | - | 0.342 | 0.045 |  |
| rs3796508 | | - | -1.000 |  |  | rs3796508 | | - | 0.010 |  |
| rs3775073 | |  | - |  |  | rs3775073 | |  | - |  |
|  |  |  |  |  |  |  |  |  |  |  |
| **TLR6** | **MKK** |  |  |  |  | **TLR6** | **LWK** |  |  |  |
| D' |  |  |  |  |  | D' |  |  |  |  |
|  | rs5743808 | rs3796508 | rs3775073 |  |  |  | rs5743808 | rs3796508 | rs3775073 |  |
| rs5743808 | - | 1.000 | 0.665 |  |  | rs5743808 | - | 1.000 | 1 |  |
| rs3796508 | | - | 0.999 |  |  | rs3796508 | | - | 0.909 |  |
| rs3775073 | |  | - |  |  | rs3775073 | |  | - |  |
|  |  |  |  |  |  |  |  |  |  |  |
| R^2^ |  |  |  |  |  | R^2^ |  |  |  |  |
|  | rs5743808 | rs3796508 | rs3775073 |  |  |  | rs5743808 | rs3796508 | rs3775073 |  |
| rs5743808 | - | 0.423 | 0.013 |  |  | rs5743808 | - | 0.122 | 0.042 |  |
| rs3796508 | | - | 0.012 |  |  | rs3796508 | | - | 0.005 |  |
| rs3775073 | |  | - |  |  | rs3775073 | |  | - |  |
|  |  |  |  |  |  |  |  |  |  |  |
| **TLR6** | **ASW** |  |  |  |  | **TLR6** | **YRI** |  |  |  |
| D' |  |  |  |  |  | D' |  |  |  |  |
|  | rs5743808 | rs3796508 | rs3775073 |  |  |  | rs5743808 | rs3796508 | rs3775073 |  |
| rs5743808 | - | 1.000 | 1 |  |  | rs5743808 | - | 1.000 | 1 |  |
| rs3796508 | | - | 1.000 |  |  | rs3796508 | | - | 0.999 |  |
| rs3775073 | |  | - |  |  | rs3775073 | |  | - |  |
|  |  |  |  |  |  |  |  |  |  |  |
| R^2^ |  |  |  |  |  | R^2^ |  |  |  |  |
|  | rs5743808 | rs3796508 | rs3775073 |  |  |  | rs5743808 | rs3796508 | rs3775073 |  |
| rs5743808 | - | 0.449 | 0.109 |  |  | rs5743808 | - | 0.196 | 0.03 |  |
| rs3796508 | | - | 0.049 |  |  | rs3796508 | | - | 0.006 |  |
| rs3775073 | |  | - |  |  | rs3775073 | |  | - |  |
|  |  |  |  |  |  |  |  |  |  |  |
| **TLR4** | **UG** |  |  |  |  | **TLR4** | **SA** |  |  |  |
| D' |  |  |  |  |  | D' |  |  |  |  |
|  | rs4986790 | rs5030719 | rs2770150 |  |  |  | rs4986790 | rs5030719 | rs2770150 |  |
| rs4986790 | - | 1.000 | 1.000 |  |  | rs4986790 | - | 1.000 | 1.000 |  |
| rs5030719 | | - | 1 |  |  | rs5030719 | | - | 1 |  |
| rs2770150 | |  | - |  |  | rs2770150 | |  | - |  |
|  |  |  |  |  |  |  |  |  |  |  |
| R^2^ |  |  |  |  |  | R^2^ |  |  |  |  |
|  | rs4986790 | rs5030719 | rs2770150 |  |  |  | rs4986790 | rs5030719 | rs2770150 |  |
| rs4986790 | - | -1.000 | 0.008 |  |  | rs4986790 | - | -1.000 | -1.000 |  |
| rs5030719 | | - | -1.000 |  |  | rs5030719 | | - | -1.000 |  |
| rs2770150 | |  | - |  |  | rs2770150 | |  | - |  |
|  |  |  |  |  |  |  |  |  |  |  |
| **TLR4** | **MKK** |  |  |  |  | **TLR4** | **LWK** |  |  |  |
| D' |  |  |  |  |  | D' |  |  |  |  |
|  | rs4986790 | rs5030719 | rs2770150 |  |  |  | rs4986790 | rs5030719 | rs2770150 |  |
| rs4986790 | - | 0.905 | 0.346 |  |  | rs4986790 | - | 0.715 | 1.000 |  |
| rs5030719 | | - | 0.638 |  |  | rs5030719 | | - | 0.531 |  |
| rs2770150 | |  | - |  |  | rs2770150 | |  | - |  |
|  |  |  |  |  |  |  |  |  |  |  |
| R^2^ |  |  |  |  |  | R^2^ |  |  |  |  |
|  | rs4986790 | rs5030719 | rs2770150 |  |  |  | rs4986790 | rs5030719 | rs2770150 |  |
| rs4986790 | - | 0.392 | 0.002 |  |  | rs4986790 | - | 0.478 | 0.016 |  |
| rs5030719 | | - | 0.004 |  |  | rs5030719 | | - | 0.004 |  |
| rs2770150 | |  | - |  |  | rs2770150 | |  | - |  |
|  |  |  |  |  |  |  |  |  |  |  |
| **TLR4** | **ASW** |  |  |  |  | **TLR4** | **YRI** |  |  |  |
| D' |  |  |  |  |  | D' |  |  |  |  |
|  | rs4986790 | rs5030719 | rs2770150 |  |  |  | rs4986790 | rs5030719 | rs2770150 |  |
| rs4986790 | - | 1.000 | 1.000 |  |  | rs4986790 | - | 0.542 | 0.983 |  |
| rs5030719 | | - | 1 |  |  | rs5030719 | | - | 0.105 |  |
| rs2770150 | |  | - |  |  | rs2770150 | |  | - |  |
|  |  |  |  |  |  |  |  |  |  |  |
| R^2^ |  |  |  |  |  | R^2^ |  |  |  |  |
|  | rs4986790 | rs5030719 | rs2770150 |  |  |  | rs4986790 | rs5030719 | rs2770150 |  |
| rs4986790 | - | 0.484 | 0.008 |  |  | rs4986790 | - | 0.227 | 0.004 |  |
| rs5030719 | | - | 0.004 |  |  | rs5030719 | | - | 0.003 |  |
| rs2770150 | |  | - |  |  | rs2770150 | |  | - |  |
|  |  |  |  |  |  |  |  |  |  |  |
| **TLR2** | **UG** |  |  |  |  | **TLR2** | **SA** |  |  |  |
| D' |  |  |  |  |  | D' |  |  |  |  |
|  | rs3804099 | rs3804100 |  |  |  |  | rs3775073 | rs3796508 |  |  |
| rs3804099 | - | 0.000 |  |  |  | rs3804099 | - | 0.000 |  |  |
| rs3804100 | | - |  |  |  | rs3804100 | | - |  |  |
|  |  |  |  |  |  |  |  |  |  |  |
| R^2^ |  |  |  |  |  | R^2^ |  |  |  |  |
|  | rs3775073 | rs3796508 |  |  |  |  | rs3775073 | rs3796508 |  |  |
| rs3804099 | - | 0.000 |  |  |  | rs3804099 | - | 0.000 |  |  |
| rs3804100 | | - |  |  |  | rs3804100 | | - |  |  |
|  |  |  |  |  |  |  |  |  |  |  |
| **TLR2** | **MKK** |  |  |  |  | **TLR2** | **LWK** |  |  |  |
| D' |  |  |  |  |  | D' |  |  |  |  |
|  |  |  |  |  |  |  |  |  |  |  |
|  | rs3804099 | rs3804100 |  |  |  |  | rs3775073 | rs3796508 |  |  |
| rs3804099 | - | 1.000 |  |  |  | rs3804099 | - | 1.000 |  |  |
| rs3804100 | | - |  |  |  | rs3804100 | | - |  |  |
|  |  |  |  |  |  |  |  |  |  |  |
| R^2^ |  |  |  |  |  | R^2^ |  |  |  |  |
|  | rs3775073 | rs3796508 |  |  |  |  | rs3775073 | rs3796508 |  |  |
| rs3804099 | - | 0.014 |  |  |  | rs3804099 | - | 0.027 |  |  |
| rs3804100 | | - |  |  |  | rs3804100 | | - |  |  |
|  |  |  |  |  |  |  |  |  |  |  |
| **TLR2** | **ASW** |  |  |  |  | **TLR2** | **YRI** |  |  |  |
| D' |  |  |  |  |  | D' |  |  |  |  |
|  |  |  |  |  |  |  |  |  |  |  |
|  | rs3804099 | rs3804100 |  |  |  |  | rs3804099 | rs3804100 |  |  |
| rs3804099 | - | 1.000 |  |  |  | rs3804099 | - | 1.000 |  |  |
| rs3804100 | | - |  |  |  | rs3804100 | | - |  |  |
|  |  |  |  |  |  |  |  |  |  |  |
| R^2^ |  |  |  |  |  | R^2^ |  |  |  |  |
|  | rs3775073 | rs3796508 |  |  |  |  | rs3775073 | rs3796508 |  |  |
| rs3804099 | - | 0.044 |  |  |  | rs3804099 | - | 0.039 |  |  |
| rs3804100 | | - |  |  |  | rs3804100 | | - |  |  |
